# Utilising Random Effects Models to Analyse Multiple Mini-Interviews for Prospective Medical Students – From Theory to Practice

**DOI:** 10.1177/23821205251411170

**Published:** 2026-01-30

**Authors:** Chezko Malachi Peligrino Castro, Nicola Phillips, Karen Grant, Iain Robinson, Emanuele Giorgi

**Affiliations:** 1 Medical School, 151569 Lancaster University Medical School, Lancaster, UK; 2 572253 Lancaster Medical School, Centre for Health Informatics Computing and Statistics, Lancaster University, Lancaster, UK

**Keywords:** multiple mini-interviews (MMIs), medical school admissions, statistical modelling, mixed effects model

## Abstract

**Background:**

Multiple-mini-interviews (MMIs) are the most commonly used non-academic assessment tool for British medical school admissions processes. Potential inconsistencies can arise from running MMIs, such as differing marking standards among interviewers and stations with varying levels of difficulty.

**Methods:**

With the aim of analysing MMI data, the cumulative probit mixed model was deployed which accounts for latent sources of variation inherent to MMI scores – both external factors, such as interviewer behaviour and station complexity, as well as the factor of interest – candidates’ true performance at interview. With the secondary aim of making this methodology more accessible to non-statistical experts, we developed a user-friendly application using R Shiny. The app was created to standardise MMI scores and generate feedback for interviewers without requiring prior knowledge of programming.

**Results:**

MMI data from Lancaster Medical School were analysed for the academic year 2022–2023. Applicant ability (*n* = 352) contributed to 22.94% of the total variance in MMI scores. Notably, interviewers (*n* = 83) contributed a smaller proportion of variance (10.79%). Station difficulty (*n* = 9) had a minor impact on variance (2.23%), with inter-station reliability being acceptable based on the Cronbach's alpha value (α = 0.7072).

**Conclusion:**

The current method provides a statistically robust approach towards the analysis of MMI scores. In addition, its companion application can be used by admissions staff to communicate feedback to interviewers on their scoring patterns and identify stations that are better at discriminating applicants’ ability. The illustrated approach can be adapted for use by other medical institutions that use MMI scores to rank candidates during their admissions processes. Future research could investigate how to extend the proposed modelling approach to address applicants’ background factors, such as socio-economic status, for more comprehensive analysis and more equitable offer allocation.

## Introduction

Traditionally, the medical school admissions process has greatly emphasised academic ability, gradually shifting towards a more holistic approach of assessing applicants’ attributes, attitudes, and skills. Within the British context, this has been accompanied in recent years by plans to increase medical school cohort sizes as per the National Health Service's Long Term Workforce Plan.^1^ To balance the need to meet escalating demand for doctors in an increasingly pressured healthcare system with the justifiable, yet expensive cost of training medical students, it falls to medical schools to develop robust assessment methods by which applicants deemed most “suitable” for medicine can be selected.

Historically, selection for medical school candidates utilised the traditional panel interview format. This has been replaced by a majority of medical schools with the MMI format, or multiple-mini-interview, which, alongside markers of academic attainment (eg, predicted grades) and performance in medical aptitude tests, such as the University Clinical Aptitude Test (UCAT) or the now-defunct BioMedical Admissions Test (BMAT), provide the basis for making offers. First used in medical admissions processes at McMaster University in 2004, MMIs consist of a series of short, objective structured clinical examination (OSCE)-style stations that allow assessment of various topics relevant to medicine, such as communication skills and knowledge of healthcare issues.^2^ Indeed, current recommendations from the Medical Schools Council favour the use of MMIs alongside academic attainment and aptitude tests for their selection processes, as stated in the Selecting for Excellence report.^3^

Within the wider literature, there is increasing evidence that MMIs have predictive validity for future academic performance, such as in national licensing examinations and OSCEs.^4–6^ However, a common flaw affecting the reliability of MMIs is a lack of control in the variation of marking behaviours between interviewers (henceforth referred to as inter-rater variability). Most medical schools conduct MMIs over several days, with simultaneous circuits, and with a wide array of interviewer backgrounds, such as academic staff, clinicians, or medical students, each with their own unconscious scoring behaviours and candidate preferences.^7,8^ As proposed by Myford and Wolfe in a 2004 paper, effects of raters (interviewers) on scores can be categorised into five groups: leniency/severity, central tendency, randomness (inconsistency), the halo effect, and differential leniency/severity (ie, restriction of range).^9^ In addition to these behaviours is the concept of “contrast effects,” where the assessed performance of one candidate is affected by the previous candidates’ performance, which has been observed in similar assessment formats.^10^ Through such mechanisms, interviewers may significantly contribute to the unexplained variance in MMIs and hence decrease the reliability of the admissions process if not properly addressed.

Several papers have utilised the multifactorial Rasch model (MFRM) or generalisability theory (G theory) in their analysis of MMI to account for variation in MMI scores.^11–13^ MFRMs provide rich information regarding the interactions between interviewer, station, and interviewee through the use of facets in analysing MMI data. While MFRMs may be statistically efficient with increasingly large pools of MMI candidates (they require a large number of observations to reliably estimate parameters), they can become time-intensive due to the need for repeated iterations in fitting observations to the models. Furthermore, the analysis and interpretation of assessment data using MFRMs require that admissions staff be proficient in the relevant statistical software program.^14^

To resolve the tension between statistical rigour and ease of use, we pursue the two following objectives: (1) To develop a statistically efficient and robust modelling approach to estimate applicants’ abilities while accounting for additional variation in MMI scores introduced by interviewers’ marking severity, station difficulty, and day of interview; and (2) To illustrate a user-friendly interface that allows for ease of use of the above method by practitioners who do not have formal training in statistics.

In meeting our primary objective, we model the MMI scores using cumulative probit mixed models (CPMMs)^15^^(pp122-123)^ by utilising random effects to model variation in candidates’ MMI scores, alongside the three sources of residual variation – interviewer, station, and day of interview. We argue that this modelling framework has two main advantages over standard approaches based on MRFMs. The first is that it is statistically more efficient as it exploits the information provided by the ordering of the scores, thus requiring the estimation of a smaller number of model parameters; in addition, the use of random effects also allows us to borrow strength of information across the data, which can help reduce uncertainty to estimate each candidate's performance.

To improve accessibility of this approach, we propose a digital application format as a solution for conducting MMI station scoring analysis. By utilising a user-friendly interface created with Shiny, an R-based application framework,^16^ both standards can be met. Previous studies have highlighted the use of Shiny applications to simplify statistical modelling in educational and psychometric assessments,^17^ but this study is the first to apply a Shiny application within the context of medical education.

## Methods

### MMI Setting and Process at Lancaster University

The selection process for the 2022–2023 admissions cycle (2023 entry) at Lancaster Medical School consisted of the following stages: candidate's UCAS applications were first screened against the academic entry requirements, then ranked according to their BMAT test results, with the higher-scoring applicants shortlisted for interview. Overseas and Gateway Year applicants were processed separately and were excluded from this analysis.

Interviews were conducted online, over a total of seven days, with duplicate MMI circuits held on each day across two months. Applicants who failed to attend the interview were excluded from the data set, leaving a final sample set of 352 interviewed applicants included in the final analysis. The sample size was naturally derived from the admissions process, which equates to a total population sampling method; to allow for comparison between potential cohorts, scaling according to total variation in interview scores was applied (see section on Statistical Modelling).

### Interviewer Selection

In total, 83 interviewers were selected from various stakeholder groups: academic staff from Lancaster Medical School, clinicians involved in medical education from primary care and hospital settings, medical students, and members of our patients and public representatives group. Prior to the interview, interviewers were asked to prepare through watching a short instruction video and reading station documentation that familiarised them with the practicalities of the interview, the station purpose, skills and attributes assessed and marking criteria. All interviewers attended training on their first day of interview, which included detailed advice regarding the use of the marking rubric and the potential impact of bias on scoring. Interviewers had time to prepare for their station and ask questions before the interview began.

To reduce inter-rater variability, interviewer backgrounds were matched with relevant MMI stations. For example, student interviewers were placed exclusively at the student-led station (where students had also developed the task, questions, and marking criteria).

### Format of MMI/Scoring Criteria

The MMI consisted of nine assessed stations, including one standalone 20-minute group discussion station (station C1) and eight 5-minute MMI stations (stations A1–4 and B2, B3, B4, B6). Each station was assessed by one interviewer. In addition to the assessed stations, the MMI circuit also contained two 5-minute preparation stations, where the applicant would be given a task to complete prior to the assessed station, two break stations and an administration station. This time was chosen to minimise the overall assessment period while maintaining the ability to differentiate between applicants and test reliability.

In each MMI station, interviewers were asked to score candidates in three domains, using a 5-point scale, creating a maximum scoring range from 3 to 15. These three domains mainly consisted of two skill domains and a global domain, indicating the interviewer's overall impression of a candidate's suitability to study medicine, given their performance in that station. Within the scale, the marking criteria associated with the mid-point score (3) represented the standard expected of a medical student at the point of entry. At this score, a student would be likely to be able to cope with the demands of a medical degree. Likewise, the high-point score (5) corresponded to an outstanding applicant whose performance was above what was expected at the point of entry, and the low-point score (1) demonstrated that an applicant's performance was below expectations and may struggle with the demands of the course.

All stations were trialled before implementation by first-year medical students and senior interviewers. Detailed descriptors for scoring candidates on each domain of assessment were standard-set during these trials, and station instructions were assessed for clarity and effectiveness. Topics assessed at different stations were taken from various content domains, testing generic attributes desirable for medical school, such as communication skills and knowledge of medical ethics principles. Scores were recorded for analysis in an Excel file, and identifiable candidate information was anonymised by use of candidate number and UCAS ID.

### Statistical Modelling

For the analysis of MMI scores, we developed a CPMM^15^^(pp122-123)^ to describe the relationship between observed scores, candidates, interviewers, day of the interview, and station difficulty.

Let *Y_ijkt_* represent the observed score of a particular candidate (*i*) from a particular interviewer (*j*) from a particular station (*k*) on a particular day (*t*). We assume *Y_ijkt_* represent the discretised version of a continuous latent variable Y*_ijkt_, which is derived from candidate ability (*S_i_*), heterogeneity in interviewers’ marking patterns (*T_j_*), station difficulty (*U_k_*), and effects based on the day of the interview (*V_t_*). Additionally, we assume that Y*_ijkt_, conditionally on *S_i_*, *T_j_*, *U_k_*, and *V_t_* is a Gaussian distribution, with a mean of μ + *S_i_* + *T_j_*+*U_k_* + *V_t_*, where μ = overall average student performance.

Therefore, we can write that:

$$P(Y_{ijkt}^{*}\;<\alpha_{h}\;|S_{i},\;T_{j},\;U_{k},V_{t})=\Phi (\alpha_{h}-[\mu +S_{i}+T_{j}+U_{k}+V_{t}]),$$

where *α_h_* are the thresholds such that an observed MMI score *Y_ijkt_* satisfies

$$\begin{array}{rl} & Y_{ijkt}=h\;\;\;\;\;\;\;\mathrm{if}\;\mathrm{and}\;\mathrm{only}\;\mathrm{if}\\ & \alpha_{h-1}<Y_{ijkt}^{*}<\alpha_{h}\;\;\;\;\mathrm{for}\;\;h=3,4,\ldots ,15. \end{array}$$

We also assume that *S_i_*, *T_j_*, *U_k_*, and *V_t_* are each independent, zero-mean Gaussian distributions, with respective variances of $\sigma_{S}^{2}$ , $\sigma_{T}^{2}$ , $\sigma_{U}^{2}$ , and $\sigma_{V}^{2}$ .

The objective in utilising this model is to make inferences based on *S_i_*, which represents the random effect of candidate ability (how much of an impact a candidate makes on the overall interview scores independent of interviewers, station, or days). Under ideal circumstances, the respective contributions of these external factors should be *T_j_ *=* U_k_ *=* V_t_ *= 0 for all *j*, *k*, and *t*. This situation is unlikely to be the case in real-world settings, and so these variables must also be accounted for to understand their relative contribution to total variation in MMI scores. Said variables are also scaled according to the variation within the uploaded dataset, such that the effect of an individual candidate's ability on their MMI scores is generalisable to all theoretical cohorts using the same stations.

A nonparametric block bootstrap method (*n* = 1000 replicates) was utilised to generate 95% confidence intervals, which would account for the nested structure of the MMI data, and median estimates for the variance components were calculated to obtain unbiased estimates of variance for the random effects components.

The analysis was undertaken by developing a Shiny R application^16^ within the R program (4.3.0),^18^ using the package “ordinal” to fit the CPMM to the MMI data.^19^ The Shiny package was used in order to create a digital interface by which admissions staff could upload a spreadsheet of candidate MMI scores and receive a ranked list of candidates based on the *S_i_*. Additional functions were also added to the app for ease of analysis, displaying *T_j_* (variation in interviewer marking behaviour) and *U_k_* (station difficulty/easiness), as well as simple graphical feedback for interviewers.

Postanalysis, candidates were first ranked by total MMI score to estimate a threshold for offer-making. The candidate list is then re-ranked based on calculated *S_i_*, and those with a lower total score but a competitive *S_i_* were made an offer to study. Candidates with an *S_i_* rank below this threshold but considered to have performed well at the interview were held on a waiting list.

## Results

### Analysis of MMI Scores

**Figure 1.**
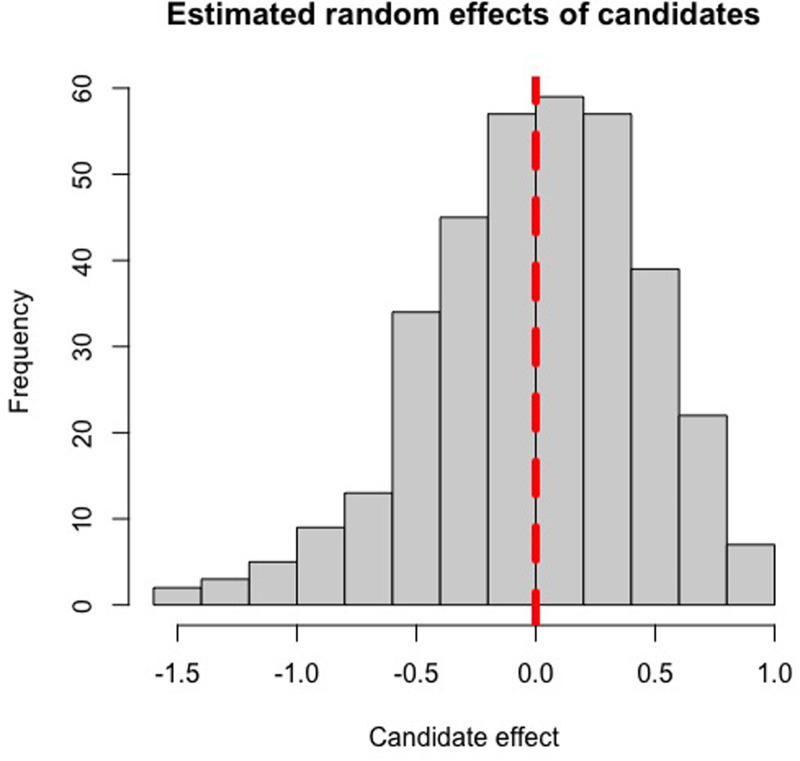
Histogram of the estimated random effects from the 2022-2023 MMI cohort, depicting the distribution of candidate ability. The vertical red line indicates a random effect of zero.

**Figure 2.**
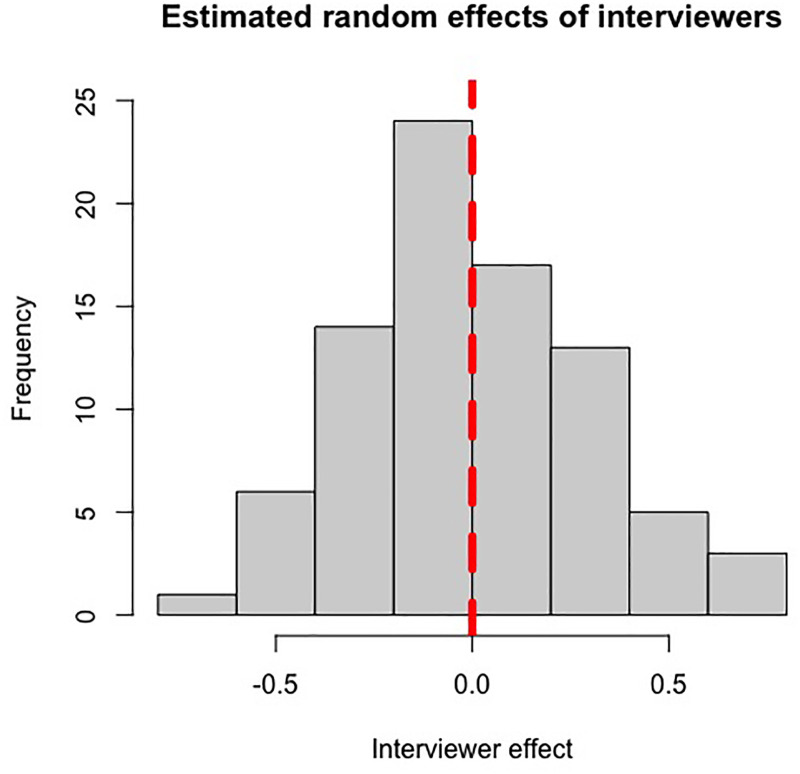
Histogram of the estimated random effects from the 2022-2023 MMI cohort, depicting the distribution of interviewer marking behaviours. The vertical red line indicates a random effect of zero.

A total of 352 candidates’ MMI scores were analysed through the CPMM method, utilising their individual random effects value to create a ranked list of candidates for offers. Candidates who performed above average at interview (independent of interviewer or station-induced effects) would have positive random effects values, and vice versa, with the magnitude of their (*S_i_*) value representing to what extent a candidate's ability at interview impacted their MMI scores in standard deviations from the mean. Based on the histograms of estimated random effects values of candidates and interviewers (Figures 1 and 2), candidate ability and interviewer severity appear to be normally distributed, confirming our prior assumptions required for the CPMM to hold.

Cronbach's alpha was calculated between stations and was found to be acceptable (α = 0.7072). In line with expectations, candidate ability contributed the most to variation based on bootstrapped estimates (22.94%); notably, interviewers marking behaviours explained a small proportion of variance in scores (10.79%). Differences due to station difficulty (2.23%) or day (0.19%) had little effect on MMI scores (Table 1). From the original model, variance estimates attributable to candidate ability and interviewer behaviour fell outside the bootstrapped interval, which suggests that the maximum likelihood estimator is biased for those variables, and therefore justifies the adoption of the more statistically robust bootstrap estimates.

**Table 1.** Estimated Variances of Random Effects and Contribution to Total Variance Within Multiple-Mini-Interview (MMI) Scores.

Variables	Variance of Random Effects from Fitted Model	Total Contribution to Variance^a^	Bootstrapped Median Estimate of Variance (95% CI)	Bootstrapped Percentage Contribution to Variance (95% CI)
Candidate ability (*n* = 352)	0.2934	20.39%	0.361 (0.2993-0.4215)	22.94% (19.83%-25.7%)
Interviewer marking behaviours (*n* = 83)	0.1164	8.09%	0.1692 (0.131-0.219)	10.79% (8.67%-13.6%)
Station difficulty (*n* = 9)	0.0255	1.77%	0.035 (0.0165-0.0585)	2.23% (1.05%-3.61%)
Day-induced effect (*n *= 7)	0.0037	0.26%	0.003 (0-0.0256)	0.19% (0%-1.67%)

95% CI by nonparametric bootstrapping provided in parentheses.

a Total variance of the original fitted model = 1.4390. Residual variance in a probit model = 1.

**Figure 3.**
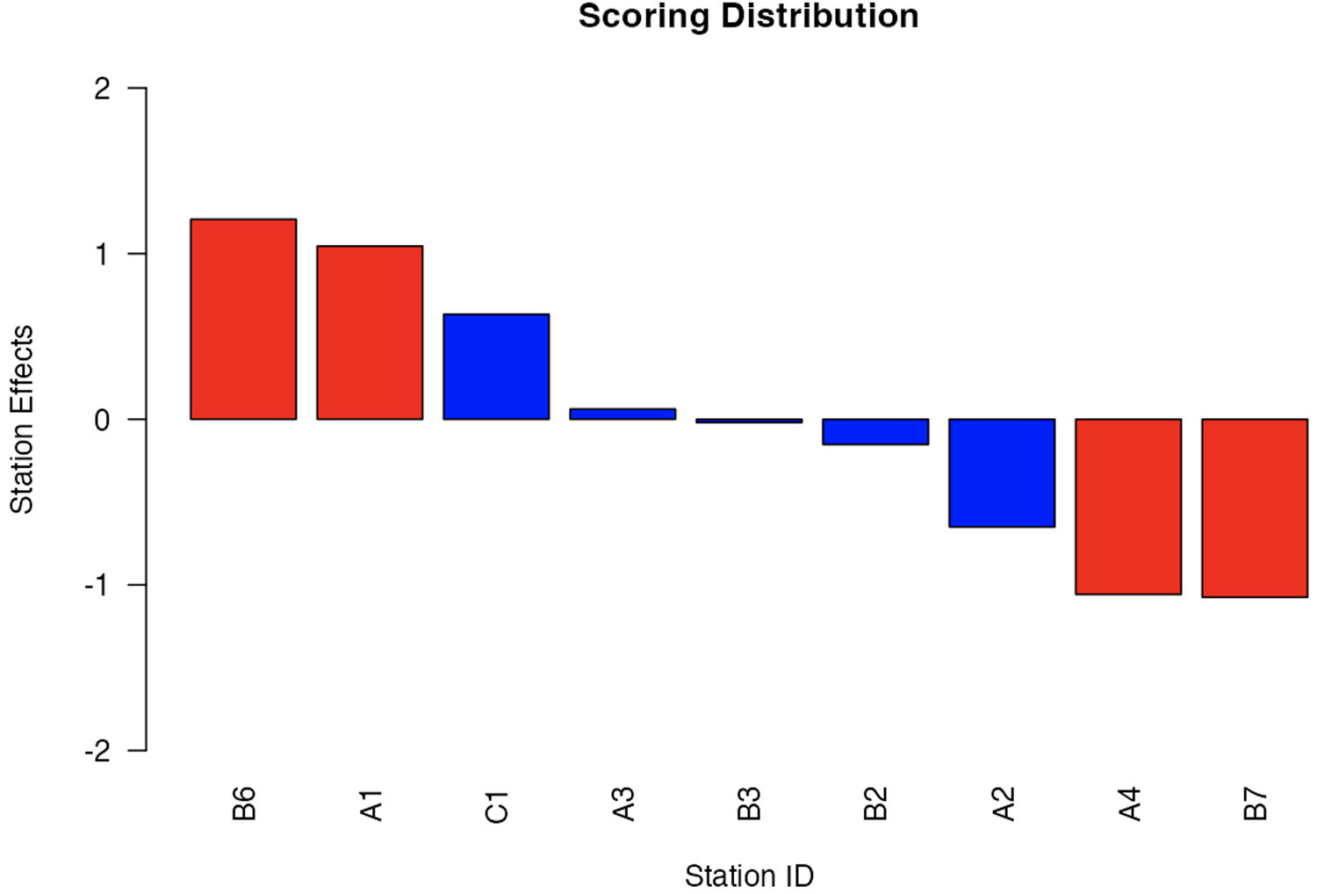
Bar chart showing the random effects of MMI stations. *Note:* The stations A1, A4, B6, and B7, coloured red, indicating random effect values at least one standard deviation above and below the mean (zero).

Particular stations were identified on analysis as having random effects greater than one, or less than minus one – for example, station B6 and station B7, with random effect values of 1.23 and −1.10, respectively (Table 2 and Figure 3). These stations were kept in the analysis due to the necessity of the attributes tested at each station. The high positive effect of B6 suggested that candidates likely found the content of the station easier, which thus had a positive impact on candidate scores overall, while station B7 was particularly difficult and negatively impacted candidate scores.

**Table 2.** Multiple-Mini-Interview stations with corresponding predictive means of the random effects.

Station ID	Station Random Effect (*U_K_*)	Station Content
A1	1.0454	Motivation to study medicine, insight into own abilities
A2	−0.6506	Effective communication (asking questions), empathy and respect for others
A3	0.0618	Empathy and ability to care for others, insight into own health
A4	−1.0573	Effective communication (giving instructions), respect for others
B2	−0.1522	Critical thinking and problem solving
B3	−0.02	Understanding of professionalism and problem solving
B6	1.2071	Ethical reasoning and respect for others
B7	−1.0749	Teamwork and insight into own abilities
C1	0.6336	Teamwork and effective communication (group station)

Different station IDs are presented, each assessing different topics.

### Application Use

The Shiny application was successfully trialled and required little computing time to fit the CPMM to the data. Upon completion, it was able to generate a ranked list of interview candidates by performance, as well as station and interviewer feedback. Simple descriptive statistics were given to interviewers based on their impact on MMI scores. The operational manual will be attached to this article as the Supplemental Material.

**Image 1.**
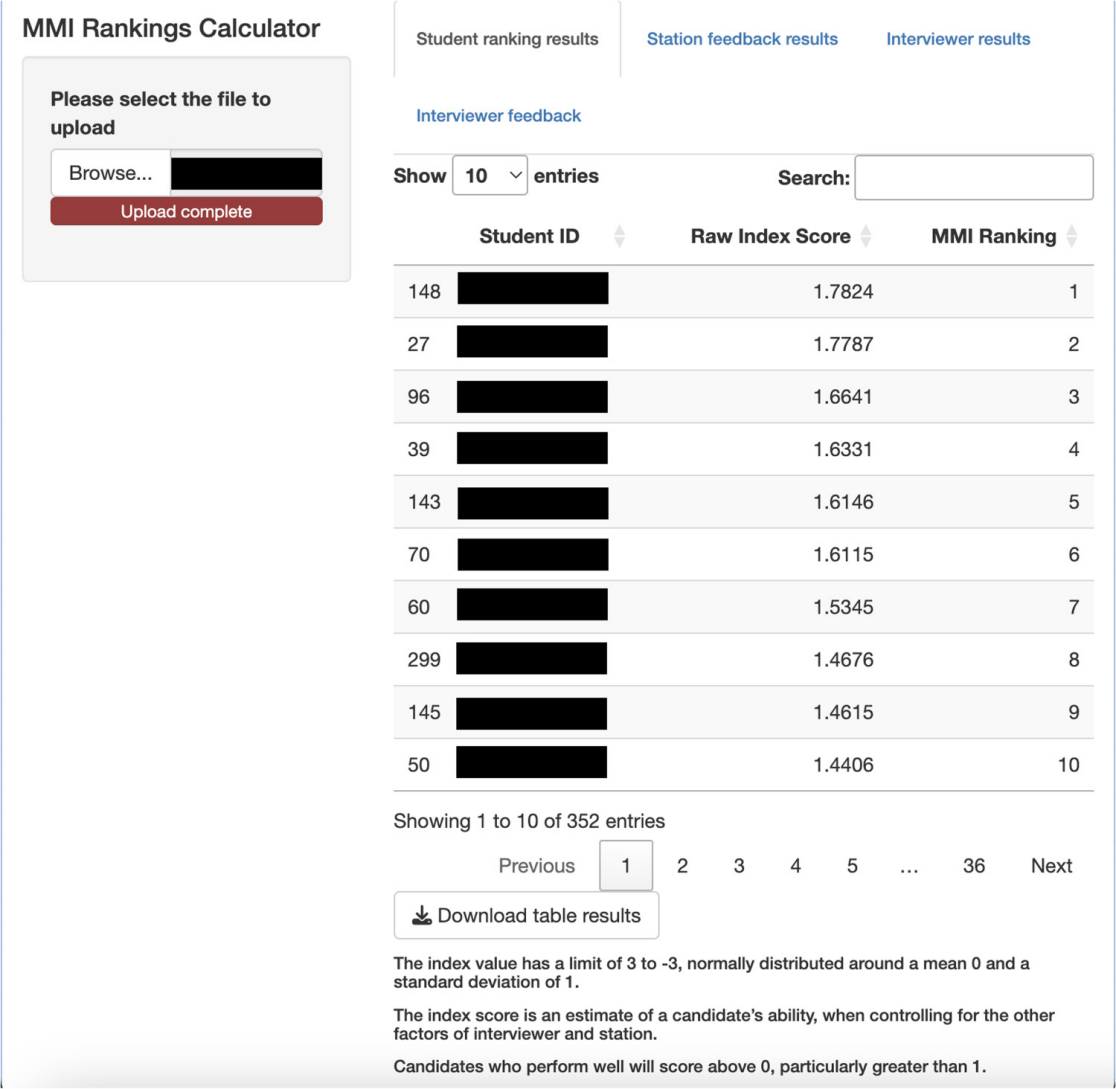
A screenshot taken from the Shiny application depicting its candidate analysis section. Identifiable information relating to candidates has been removed to protect confidentiality and privacy.

**Image 2.**
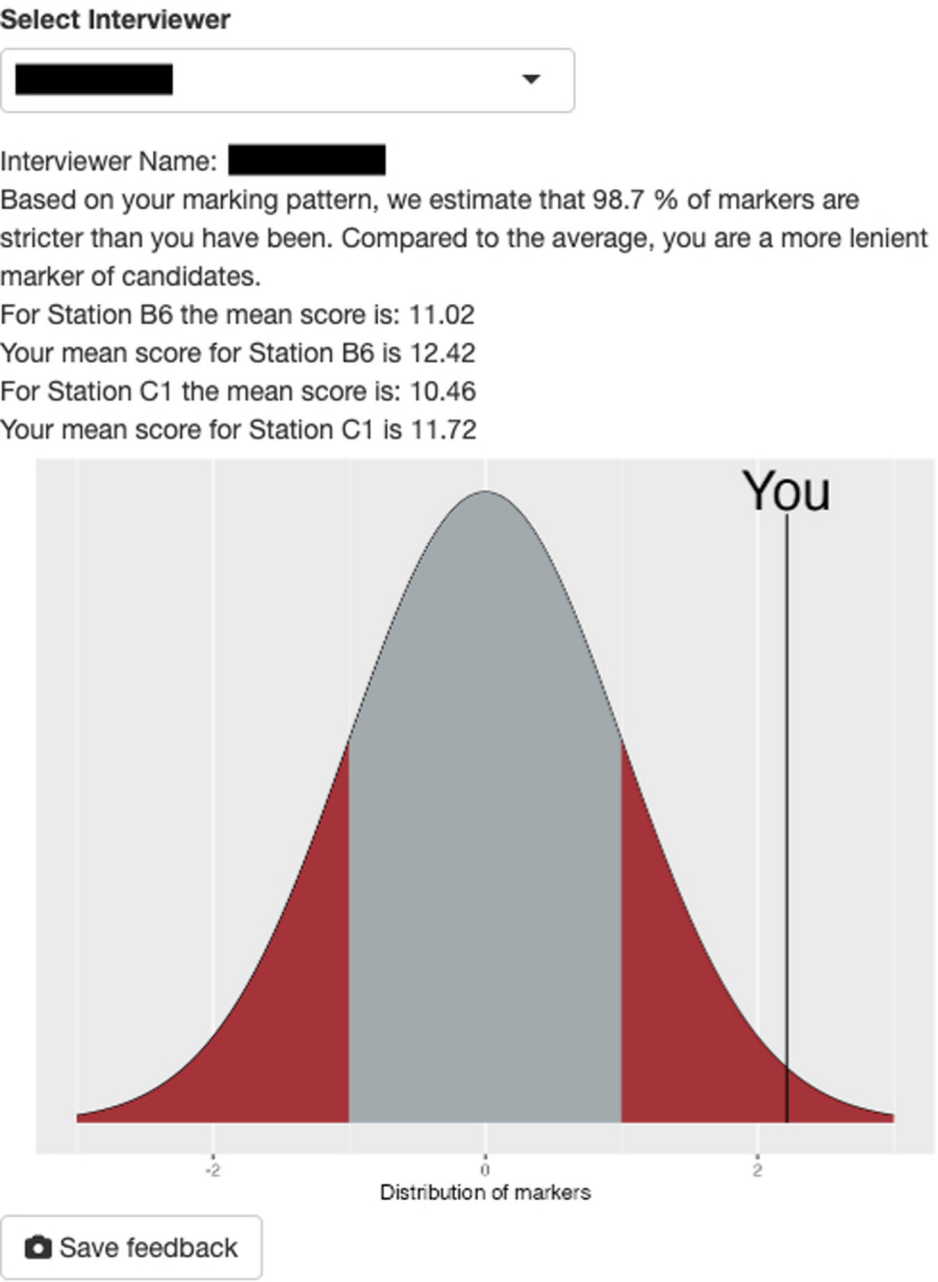
An example screenshot of basic interviewer feedback based on results calculated by the mixed effects model. The interviewer name has been removed to protect confidentiality and privacy.

Images 1 and 2 display screenshots of the Shiny application used for the MMI score analysis. Image 1 depicts the candidate ranking table generated by the application after the relevant file was uploaded and analysed. As stated previously, candidates with high random effects values were determined to have high performance at interview. Image 2 is a screenshot from the same application, showing feedback given to an individual interviewer.

## Discussion

The CPMM approach is successful at creating a sound basis for an offer allocation system, as it partitions effects attributable to specific variables, including external variables that can increase variation in candidates’ performance at interview. As expected, we found that candidates’ ability at interview explained a considerable proportion of variance in MMI scores. This is consistent with the wider body of literature which utilise MFRMs or G theory in their analyses. Such studies show similar estimates of applicant-attributable variance – Roberts et al^20^ and Till et al^21^ report an attributable percentage to student ability of 19.1% and 16.01%, respectively. For both papers, the total attributable proportion of variance (eg, to candidates or interviewers) by the Rasch model is less than the proportion of residual variance, and this article reports similar findings. However, there must be caution drawn in comparing estimates of attributable variance between MFRMs and CPMMs, as these models allocate variance via differing statistical approaches and are thus not necessarily equivalent in interpretation.

The CPMM approach also allows for the scrutiny of MMI station design. Whilst this variable did not take a large proportion of variance, the probit model still allowed for the identification of stations that tended towards the extremes of difficulty for the average medical applicant cohort. To improve future analyses, marks per station could be subdivided into the assessed domains, such that rather than stations, “topics assessed” would be the random effects variable. Additionally, this would help to unmask measurement error (eg, if the global performance mark produced undue weighting on candidates’ MMI scores).

In quantifying the external random effects variables, this method can be simultaneously deployed to identify problematic interviewer patterns – in particular, heterogeneity in interviewer marking behaviour. This may be due to a multitude of reasons, which cause consistent differences in an interviewer's allocation of marks, such as interpretations of the marking rubric or personality mismatches; however, we suggest this may be driven primarily by interviewer tendencies towards severity or leniency. Within the 2022 cohort, a tenth of overall variance was due to marking behaviour – in keeping with the extant literature, as this variable was found to be the second-largest source of explainable variance in MMI scores in papers using the MFRM method.^20,21^ Similarly, the interstation variability (Cronbach's alpha) is also in keeping with other studies utilising MFRM.^12^ The Cronbach's alpha calculated is slightly above the threshold for acceptability (>0.7). However, it must be noted that higher values of Cronbach's alpha are unlikely to be expected due to the range of topics assessed within MMI stations at Lancaster, as well as the fact that multiple domains may be assessed within a station.

### Strengths and Limitations

Unlike MFRMs, the CPMM provides a data-driven way of addressing unobserved sources of variation, making fuller use of the information in the scores and offering a practical framework for medical student selection. It is also statistically more efficient, as fewer parameters need to be estimated. At the same time, the model's ability to capture rater effects could be strengthened. While it accounts for general differences in marking behaviour, extensions to model interviewer–candidate interactions would allow it to address issues such as differential leniency or severity more explicitly. From the current specified model, the absence of applicant and interviewer background characteristics means that different sources of variation are absorbed into the random effects. In future studies, it would be worthwhile to incorporate covariates that capture relevant aspects of a candidate's background, such as socioeconomic status or prior exposure to healthcare work experience. Similarly, interviewer characteristics could also be modelled explicitly. This would reduce reliance on the random effects, leading to smaller residual variation and consequently less uncertainty in the estimates, as the random effects are shrunk closer to zero when covariates explain more of the variation. While there is the potential for observable characteristics to cause bias within the MMI, especially if omitted in modelling, the literature produces mixed results as to whether MMIs are biased against protected characteristics and socioeconomic status.^22^ Regardless, the CPMM is flexible in terms of what additional sources of variation it could account for, potentially allowing for fairer offer allocation.

While CPMMs provide a natural framework for modelling MMI scores as ordered categorical outcomes, their performance is influenced by the fineness of the MMI score scale. When the outcome has a relatively large number of categories, the cumulative probit formulation provides a good approximation to an underlying continuous latent scale, and the Gaussian assumption for the latent variable is reasonable. However, when the number of categories is small, the information content of the outcome is limited, and the proportional odds (or probit link) assumption may be less reliable in practice. In such cases, the random-effects variance can be weakly identified.

Another point that must be addressed is that the selection of interviewers and station content was designed to balance assessor roles and backgrounds while ensuring rigorous assessment. This implies that the interviewer pool is not a random sample from a wider population, and therefore, the generalisability of inferences to other institutions with different interviewer compositions may be limited. Within our institution, however, interviewer selection remains relatively stable across years, which mitigates concerns regarding internal validity. Future applications of CPMMs could strengthen confidence in the approach by adopting more elaborate hierarchical structures for modelling interviewer effects. For example, interviewers could be grouped by assessor role or professional background, allowing the model to account for similarities within these subgroups while still estimating individual-level variation. Such hierarchical specifications reduce the risk of attributing too much unexplained variation to individual interviewers, improve precision through partial pooling, and provide more interpretable variance components that reflect the different sources of heterogeneity in the assessment process. Validation across multiple cohorts and institutions would further test the robustness of these models, while repeated use within the same institution could track the stability of subgroup and individual interviewer effects over time.

Based on our institution's experience of MMI analysis using CPMMs over the last few years, we have found this approach to be a statistically robust and accessible tool in our medical admissions process. Areas for further research include further use of the model in scrutinising the effects of applicant demographics and aberrant marking behaviour on MMI scores, as well as the application of the model within other institutions.

## Conclusion

By utilising a CPMM approach in the analysis of MMI scores, we present a statistically robust method of selectively capturing candidates’ ability to perform at interview, which, through a digital application, allows for ease of analysis within admissions processes by creating a basis for a ranked offer system. This modelling method allows for the detection of deviations in interviewer marking behaviour and station easiness/difficulty through its estimation of random effects variables, which would otherwise impact MMI scores. The majority of variance in MMI scores was primarily due to variation in candidate ability, with a smaller proportion attributable to interviewer marking behaviour. Further research should focus on extending the CPMMs by incorporating interviewer-specific information, such as their level of training and historical marking behaviour, to enhance the robustness of standardisation. By refining the model to account for these factors, the approach can better mitigate variations in interviewer severity or leniency, further strengthening the reliability of MMI scoring. The proposed analytical approach to MMIs has enabled admissions staff to provide informed choices regarding offers by limiting the amount of undue influence external variables have on a candidate's MMI score.

## Supplemental Material

sj-docx-1-mde-10.1177_23821205251411170 - Supplemental material for Utilising Random Effects Models to Analyse Multiple Mini-Interviews for Prospective Medical Students – From Theory to Practice Supplemental material, sj-docx-1-mde-10.1177_23821205251411170 for Utilising Random Effects Models to Analyse Multiple Mini-Interviews for Prospective Medical Students – From Theory to Practice by Chezko Malachi Peligrino Castro, Nicola Phillips, Karen Grant, Iain Robinson and Emanuele Giorgi in Journal of Medical Education and Curricular Development

sj-R-2-mde-10.1177_23821205251411170 - Supplemental material for Utilising Random Effects Models to Analyse Multiple Mini-Interviews for Prospective Medical Students – From Theory to Practice Supplemental material, sj-R-2-mde-10.1177_23821205251411170 for Utilising Random Effects Models to Analyse Multiple Mini-Interviews for Prospective Medical Students – From Theory to Practice by Chezko Malachi Peligrino Castro, Nicola Phillips, Karen Grant, Iain Robinson and Emanuele Giorgi in Journal of Medical Education and Curricular Development

sj-docx-3-mde-10.1177_23821205251411170 - Supplemental material for Utilising Random Effects Models to Analyse Multiple Mini-Interviews for Prospective Medical Students – From Theory to Practice Supplemental material, sj-docx-3-mde-10.1177_23821205251411170 for Utilising Random Effects Models to Analyse Multiple Mini-Interviews for Prospective Medical Students – From Theory to Practice by Chezko Malachi Peligrino Castro, Nicola Phillips, Karen Grant, Iain Robinson and Emanuele Giorgi in Journal of Medical Education and Curricular Development
