# Supplementary material for: Utilising Random Effects Models to Analyse Multiple Mini-Interviews for Prospective Medical Students – From Theory to Practice
Source: J Med Educ Curric Dev. 2026 Jan 30;13:23821205251411170. doi: 10.1177/23821205251411170 (PMC12858787; doi:10.1177/23821205251411170)

**Lancaster Admissions research – applicant data and consent**

All applicants who are interviewed are given a survey to complete: <https://lancasteruni.eu.qualtrics.com/jfe/preview/previewId/c71cc073-fdb6-4bd7-b7c4-2205870df0a7/SV_3PAXBhn13drkHoa?Q_CHL=preview&Q_SurveyVersionID=current> from 2022/23

If you enter some information about your details, the second page of the survey ask about consent to use data. It says the following:

**Applicant Consent Form**

Throughout the selection process, Lancaster Medical School will gather and store data about you, including the information on your UCAS application form, your BMAT score, and your performance at the admissions multiple mini interview. This list is not exhaustive. This data is collected as part of the routine administration of the selection process for the medical degree programme.

We use this data for a number of purposes: to monitor the reliability and validity of our selection processes; to evaluate the impact of new multiple mini interview stations; to identify factors about applicants that predict success at medical school or to ensure that our selection processes do not disadvantage any particular groups within our applicant pool. Again, this list is not exhaustive.

From time to time, we may want to publish the results of such analysis to help inform the Medical Education community more widely and the purpose of this form is to ask for your consent to use this data for research and publication purposes. The data would all be completely anonymous and would not include any of your own personal details. The data would be presented in graphical or table form, or as summary data. It will not be possible to identify individual applicants from the published data.

It is entirely up to you whether you consent to the use of your data or not. Consenting to the use of your data or deciding that you would rather that your data was not used as outlined above will not have any impact on your application. Lancaster Medical School will not treat you any differently and it will not affect the outcome of your application in any way.

Please feel free to discuss this matter with your parents, teachers and friends before deciding about whether or not you are willing for Lancaster Medical School to use your data as outlined above.

If you have any questions about the use of your data, you can either email us (ugamedicine@lancaster.ac.uk) or you will have an opportunity to ask questions on the day of your interview.

The data will all be completely anonymous and will not include any of your own personal details. It will not be possible to identify individual applicants from the published data. The proposed data may include any data that is routinely collected and held as part of the administration of the selection process to the medical degree programme. All data will be held securely to ensure confidentiality of applicant information.

Deciding that you would rather that your data was not used as outlined in the attached information will not prejudice your application. Lancaster Medical School will not treat you any differently and it will not affect the outcome of your application in any way.

Applicants can change their minds and withdraw their consent up until the point when data has been submitted for publication. To withdraw your consent, please contact Dr Nicola Phillips (n.phillips1@lancaster.ac.uk) or Sam Porter (s.porter@lancaster.ac.uk ).


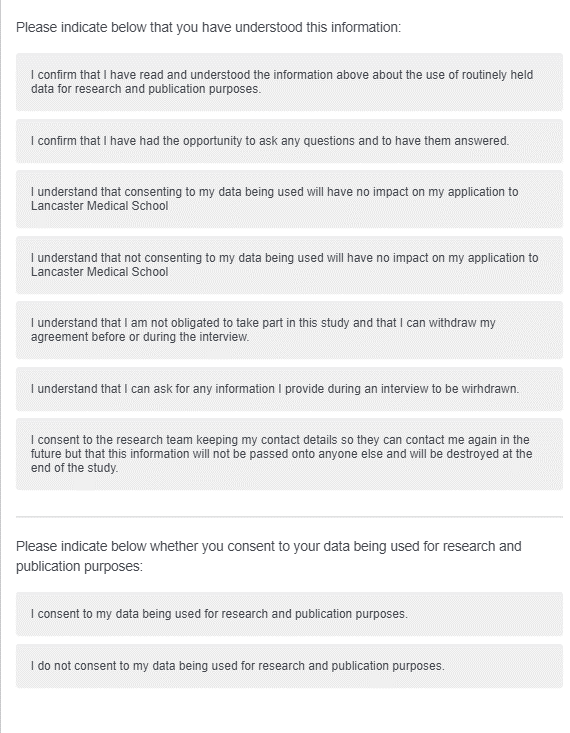

Supplement: sj-docx-3-mde-10.1177_23821205251411170 - Supplemental material for Utilising Random Effects Models to Analyse Multiple Mini-Interviews for Prospective Medical Students – From Theory to Practice [file sj-docx-3-mde-10.1177_23821205251411170.docx]
